# Teleconnection from Arctic warming suppresses long-term warming in central Eurasia

**DOI:** 10.1126/sciadv.adq9461

**Published:** 2025-03-19

**Authors:** Hainan Gong, Lin Wang, James A. Screen, Wen Chen, Judah Cohen, Renguang Wu

**Affiliations:** ^1^ Key Laboratory of Earth System Numerical Modeling and Application and Center for Monsoon System Research, Institute of Atmospheric Physics, Chinese Academy of Sciences, Beijing, China.; ^2^ Department of Mathematics and Statistics, University of Exeter, Exeter, UK.; ^3^ Department of Atmospheric Sciences, Yunnan University, Kunming, China.; ^4^ Atmospheric and Environmental Research Inc., Lexington, MA, USA.; ^5^ Department of Civil and Environmental Engineering, Massachusetts Institute of Technology, Cambridge, MA, USA.; ^6^ School of Earth Sciences, Zhejiang University, Hangzhou, China.

## Abstract

Whether the rapid warming of the Arctic, particularly the Barents-Kara Sea (BKS), substantially affects the Eurasian winter climate has been debated for over a decade. Here, we use an extended dynamical adjustment method to separate the effects of internal dynamics and thermodynamically forced BKS warming on atmospheric circulation, relying solely on observations. Evidence shows that the observed link between BKS warming and Eurasian cooling is influenced by both atmospheric internal variability and forced BKS warming. Internal variability, particularly the Arctic Oscillation, predominantly contributed to the observed Eurasian cooling from 1991 to 2012. While BKS warming has a weaker impact on Eurasian cooling on interannual to interdecadal timescales, it notably affects multidecadal scales, contributing to the observed “warming hole” in central Eurasia during 1980–2022. Our findings suggest a weak but non-negligible Eurasian cooling response to BKS warming on multidecadal timescales. These findings advance the understanding of the complex causal relationships between Arctic and mid-latitude climates.

## Teaser

Internal dynamics and BKS warming both play crucial roles on Eurasian winter climate over different timescales.

## INTRODUCTION

Over the past decades, the Arctic has experienced warming at a rate three to four times the global mean, a phenomenon referred to as Arctic amplification (AA) (*1*–*6*). In winter, the Arctic is warming especially fast in the Barents-Kara Sea (BKS) region (*7*). Accompanying the BKS warming, surface air temperature (SAT) in central Eurasia experienced sustained cooling for approximately 20 years, from the early 1990s to the early 2010s, exhibiting a pronounced warm Arctic–cold Eurasian (WACE) SAT trend pattern (*7*–*9*). Previous studies attributed cooling over central Eurasia to BKS warming associated with the sea-ice loss (*7*–*16*). It is argued that faster warming in the BKS than the lower latitudes reduces the meridional temperature gradient and potentially weakens zonal winds, inducing an anomalous anticyclonic response in the BKS and Ural regions. This shift potentially increases the possibility of cold winters over Eurasia (*7*–*16*).

Despite these findings, the causal linkage between BKS warming and Eurasian cooling remains debated (*17*–*40*). Numerous studies attribute interdecadal cooling trends in central Eurasia to internally generated anticyclonic circulation in the BKS and Ural regions, noting that climate models often underestimate the atmospheric circulation and mid-latitude cooling responses to the observed sea-ice reduction in the BKS (*17*–*35*). This inconsistency may be due to not only model deficiencies (*28*, *29*) but also a misunderstanding of statistical inference (*36*, *37*). As of now, the causal relationship between BKS warming and Eurasian cooling has yet to be conclusively resolved. The core issue lies in determining whether the observed WACE pattern is primarily driven by atmospheric internal variability, directly influenced by BKS warming, or a combination of both factors. Notably, both hypotheses point to critical anticyclonic circulation anomalies located in the BKS and Ural regions. This geographic overlap indicates that straightforward statistical analysis of observational data alone may not be sufficient to discern whether the Arctic–mid-latitude linkage results from internal dynamics or external BKS warming influences. Hence, effective approaches to separate the mid-latitude impacts of BKS warming from internal climate variability are essential to resolve the debate about the causality of the Arctic–mid-latitude linkage.

Using bespoke model experiments is one approach (*41*), but it is reliant on models being able to capture the relevant processes. Here, we introduce a complementary approach that can be applied to the observations called the extended dynamical adjustment approach (EDAA) to decompose the BKS SAT changes into winter atmospheric circulation–induced and residual thermodynamically induced components associated with anthropogenic forcings (Materials and Methods). By diagnosing the direction of surface turbulent heat flux (THF) and the changes in the preceding sea-ice concentration (SIC), we further validate the effectiveness of EDAA and support the inferred causality of the EDAA decomposition. We interpret the thermodynamically forced component as being a causal response to BKS warming (i.e., triggered by anomalous upward surface heat fluxes over the BKS during its positive phase), accompanied by the preceding SIC changes, and the winter atmospheric circulation–induced component as being a consequence of atmospheric internal variability that is a driver of, and not a response to, BKS SAT change (i.e., causing the anomalous downward surface heat fluxes over the BKS during its positive phase), without accompanied preceding SIC changes.

## RESULTS

**Fig. 1.**
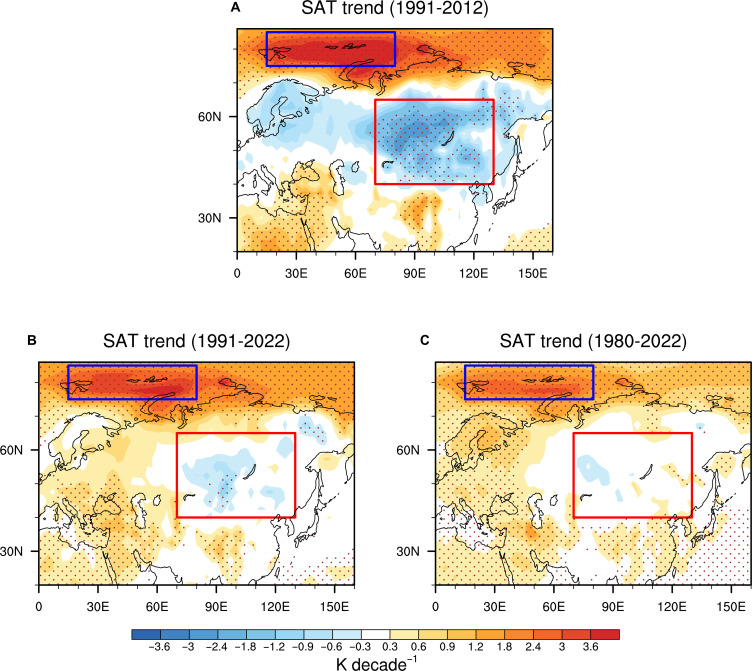
Winter (December to February) SAT changes. Winter SAT trends during 1991–2012 (**A**), 1991–2022 (**B**), and 1980–2022 (**C**) in ERA5. Dots indicate SAT trends exceeding the 95% confidence level. The blue and red boxes are the regions to define the SAT indices in the BKS and central Eurasia (CE), respectively.

Compared to the pronounced WACE pattern observed during the 1991–2012 period, BKS warming and central Eurasian cooling have weakened after 2012 despite continuous greenhouse gas forcing (Fig. 1, A and B), implying an important role of internal climate variability in the BKS warming, as well as the formation of WACE pattern. However, we also find that the winter SAT trends in central Eurasia are largely suppressed compared to surrounding areas over a long timescale (1980–2022), accompanied by the sustained BKS warming caused by greenhouse gas forcing, exhibiting a phenomenon of a “warming hole” in the Eurasian continent (Fig. 1C). This implies that the sustained warming in the BKS region may have an impact on the central Eurasian cooling on long-term timescales to some extent.

**Fig. 2.**
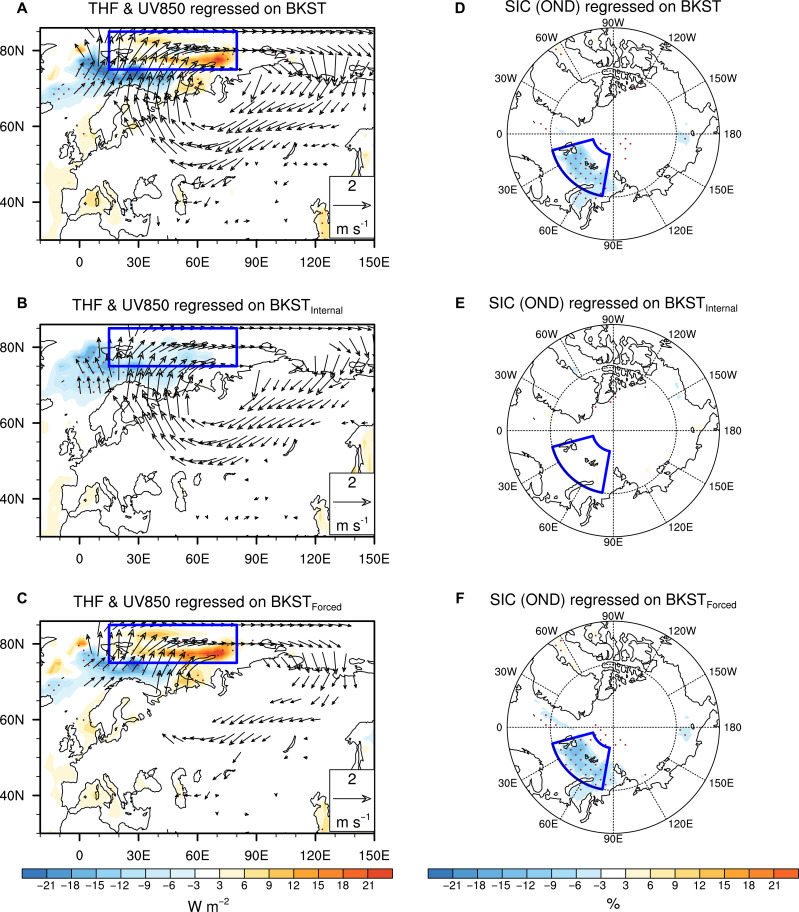
Observed changes in 850-hPa wind, THF, and preceding SIC associated with the original and decomposed SAT variability in the BKS region. (**A**) Winter 850-hPa wind (vectors) and THF (shading) anomalies regressed on the BKST index. (**B** and **C**) As in (A), but for BKST_Internal_ and BKST_Forced_ indices, respectively, during 1980–2022. (**D** to **F**) As in (A) to (C), but for the preceding late-autumn/early-winter (October to December) SIC anomalies over Arctic. Dots indicate the regions of THF and SIC changes exceeding the 95% confidence level. The wind vectors are shown only when they exceed the 95% confidence level. The BKS region is indicated by blue box.

**Fig. 3.**
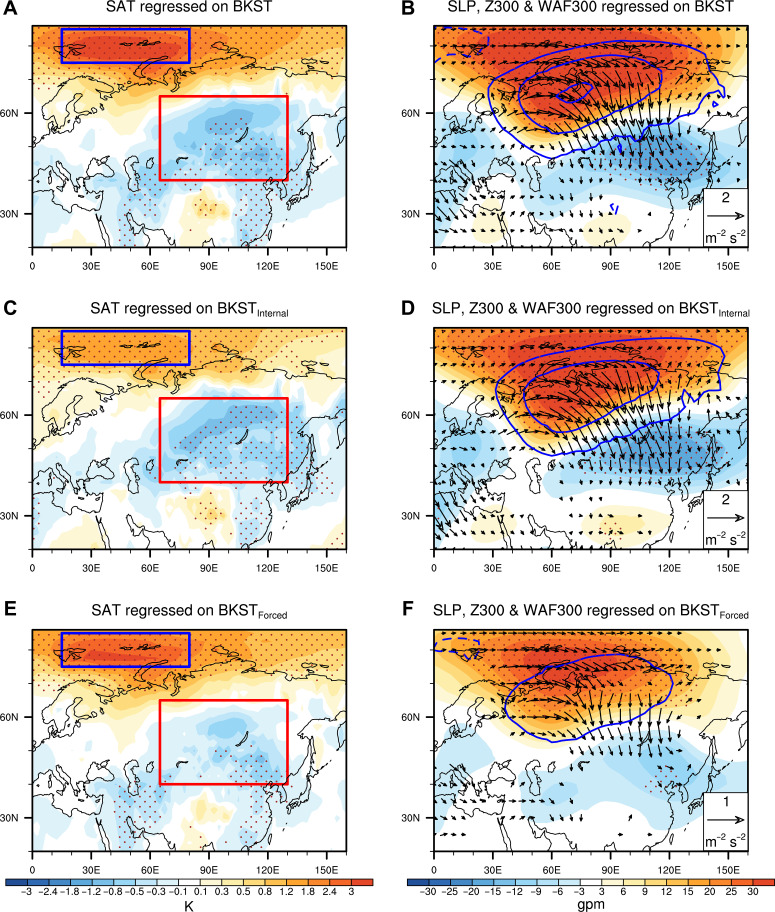
Observed changes in winter SAT and atmospheric circulation associated with the original and decomposed SAT variability in the BKS region. Winter SAT (**A**), sea level pressure (SLP) (contours, 1-hPa interval), 300-hPa geopotential height (Z300, shading), and corresponding wave activity flux (WAF300, vectors) (**B**) anomalies regressed on the BKST index during 1980–2022. (**C** and **E**) As in (A), but for BKST_Internal_ and BKST_Forced_ indices, respectively. (**D** and **F**) As in (B), but for BKST_Internal_ and BKST_Forced_, respectively. Dots indicate regions of SAT and Z300 changes exceeding the 95% confidence level. The wind vectors are shown only when they exceed the 95% confidence level. The BKS and CE regions are indicated by blue and red boxes, respectively.

To elucidate the causal link between BKS warming and central Eurasian cooling, here we define the area-averaged SAT in the region with the largest warming over BKS (75°N to 85°N, 15°E to 80°E) to represent the BKS SAT variability, denoted as BKST, and the atmospheric internally induced and thermodynamically forced (i.e., SIC-related) BKST components are denoted as BKST_Internal_ and BKST_Forced_, respectively. BKST_Internal_ displays evident interannual and interdecadal fluctuations, whereas BKST_Forced_ exhibits a pronounced warming trend during 1980–2022 (fig. S1). An anomalous anticyclone over the BKS and Ural regions, accompanied by upward THF and reduced preceding late-autumn/early-winter (October to December) SIC anomalies in the BKS regions and downward THF in the south of the BKS, is observed with the increase in BKST (Fig. 2, A and D). The wind anomalies on the west and east sides of the anticyclone transport warm and moist air from the south into the BKS and cold air from the Arctic to central Eurasia, forming the WACE pattern (Fig. 3A). This meridional dipole structure of temperature changes extends into the upper troposphere and is accompanied by weakened zonal winds over Ural regions from the surface to the upper troposphere (fig. S2A). Meanwhile, a clear Rossby wave train is observed from the Arctic to mid-latitudes in the upper troposphere (Fig. 3B). The negative geopotential height anomalies over East Asia deepen the East Asian trough and cause the widespread cooling from central Eurasia to East Asia (Fig. 3, A and B).

The atmospheric circulation, THF, and SAT anomalies associated with BKST_Internal_ resemble those associated with the original BKST (Figs. 2B and 3, C and D, and fig. S2B). But there are only anomalous downward THF over the BKS and no preceding SIC and circulation anomalies observed in the Arctic associated with the increase of BKST_Internal_ (Fig. 2, B and E). This confirms that BKST_Internal_ is winter atmospheric internally driven variability that correlates with BKST, which can contribute to the Eurasian cooling and partly BKS warming through strengthening of the southward cold advection to central Eurasia and poleward warm advection to BKS, respectively (Figs. 2B and 3C). This finding supports the previous studies that the winter internal variability plays a dominant role in the formation of WACE pattern at interannual timescales (*20*–*26*). Nevertheless, with the increase of BKST_Forced_, there are strong upward surface THF anomalies in the BKS region accompanied by the reduced preceding SIC there (Fig. 2, C and F). This supports that BKST_Forced_ essentially associated with the changes of preceding SIC. The atmospheric circulation responses to BKST_Forced_ variability somewhat resemble those associated with BKST_Internal_, but with a weaker magnitude than that related to BKST_Internal_ (Fig. 2C and fig. S2C). A Rossby wave train from the BKS to East Asia, accompanied by the central Eurasian cooling associated with increase of BKST_Internal_, is also observed, but with weaker magnitudes than that related to BKST_Internal_ (Fig. 3, E and F). This result supports the previous finding that strong BKS warming associated with the reduced preceding SIC can drive an anticyclonic response in the BKS and Ural region and influence the central Eurasian SAT changes (*7*–*9*, *12*, *14*) (Figs. 2C and 3E). This result also indicates that the observed strong connection between BKS warming and Eurasian cooling obtained through direct statistical analysis actually includes the influence of both atmospheric internal variability and thermodynamically forced, especially SIC-related BKS warming.

**Fig. 4.**
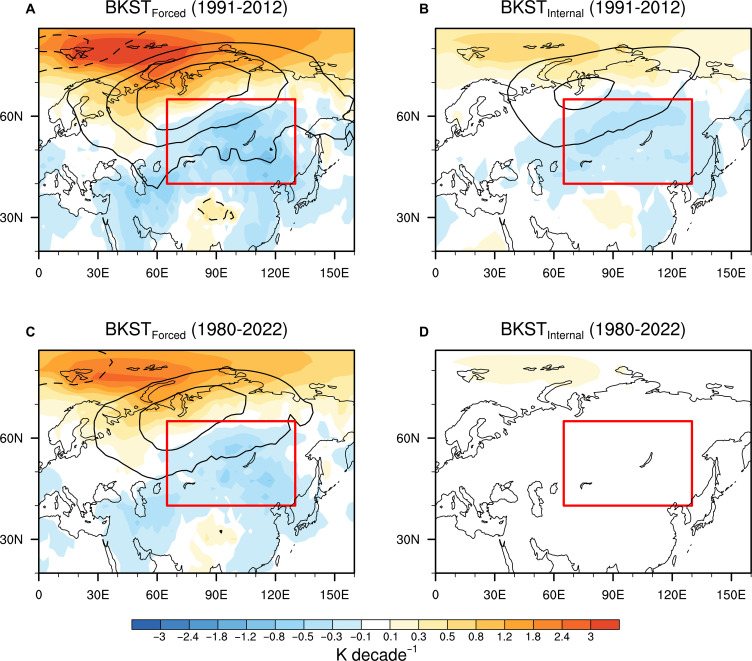
Contributions of decomposed BKST variability to the short-term and long-term atmospheric circulation and SAT trends over CE. (**A**) BKST_Forced_-caused winter SLP (contours, 0.6 hPa per decade interval) and SAT (shading) trends over Eurasian continent over the period 1991–2012. (**B**) As in (A), but for BKST_Internal_. (**C** and **D**) As in (A) and (B), respectively, but for the period 1980–2022. The CE region is indicated by red box.

On the basis of the above results, we further quantify the contribution of BKST_Internal_ and BKST_Forced_ to central Eurasian SAT changes during 1991–2012 when strong central Eurasian cooling was observed. The observed central Eurasian winter SAT (CET, 40°N to 65°N, 65°E to 130°E) trend in the period 1991–2012 is −1.02 K per decade. The BKST_Internal_ variability contributes 23.5% of CET cooling (−0.24 K per decade) in the period 1991–2012 (Fig. 4B). Note that although the BKST_Forced_ variability has a weak impact in the CET changes at the interannual scale, the rapid BKS warming during 1991–2012 (Fig. 1A) can partly compensate for its weak influence and contribute approximately 35.3% of observed CET cooling trend (−0.36 K per decade) from 1991 to 2012 (Fig. 4A). Therefore, this detectable impact of BKS warming on CET changes on interdecadal periods depends largely on the magnitude of thermodynamically forced BKS warming. Given that the overall BKST variability explains less than 60% of the observed CET cooling during 1991–2012, there must be internal variability independent of BKST variability that also plays an important role in CET changes.

It is evident that the spatial structure of atmospheric circulation anomalies associated with the BKS warming–independent CET index (CET_RM_BKST_) exhibits a pattern similar to the Arctic Oscillation (AO) (*42*) (fig. S3A). The correlation coefficient between the components in CET_RM_BKST_ and the AO index reaches 0.80 during 1980–2022 (fig. S4). This implies that the CET variability independent of BKS warming mainly arises from AO variability. Note that this AO-like circulation is unrelated to the preceding SIC forcing (fig. S3B), confirming its internal origins. During 1991–2012, the interdecadal transition of the AO from its positive to negative phase (fig. S4) reflects an anomalous anticyclone over the BKS and Ural regions and resultant widespread cooling over Eurasian continent, which accounts for approximately two-thirds of the observed CET cooling (−0.66 K per decade) in the period 1991–2012 (fig. S3C). Note that both BKST_Internal_ variability and the AO are atmospheric internal variabilities and are nearly independent of each other (*r* = −0.08 in 1980–2022). Therefore, the total winter atmospheric internal variability actually contributed to nearly 88.2% (−0.90 K per decade) of the CET cooling trend during 1991–2012. This result supports the previous studies that the observed Eurasian cooling during 1991–2012 is largely internally driven (*20*–*26*). On the basis of the observed CET trend and circulation-induced CET changes—including total BKST-related and AO-induced CET trends—from 1991 to 2012, we estimate that radiative warming driven by anthropogenic forcings contributed approximately 0.24 K per decade to CET during this period, which is broadly consistent with the best estimate of the surface warming rate due to anthropogenic forcings in Intergovernmental Panel on Climate Change Sixth Assessment Report (IPCC AR6) [~0.2 K per decade, (*43*)]. Additionally, considering that the observed phase transition of atmospheric internal variability is hard for the climate models to reproduce, this finding may partly explain why the atmospheric circulation and central Eurasian cooling responses to the fixed SIC changes in the numerical models are much weaker compared with the observed results during the period 1991–2012 (*27*, *33*).

Although the BKST_Forced_ variability exerts a relatively weaker influence on the Eurasian cooling than the internal variability at interannual and interdecadal timescale, it possibly emerges more strongly in longer-term trends because the internal variability may get averaged out over longer time period, which increases the relative contribution of BKST_Forced_ component. In the period 1980–2022, the observed CET trend is suppressed compared to its surrounding areas, with only 0.06 K per decade (Fig. 1C). There are no evident atmospheric circulation trends associated with winter internal variability, including the BKST_Internal_ and AO variability during 1980–2022 (Fig. 4D and fig. S3D), suggesting the negligible contribution of internal variability to the CET trend on long-term timescales. In contrast, BKST_Forced_ can contribute about −0.24 K per decade cooling in central Eurasia, which effectively suppresses the external radiative forcing–induced warming there (Figs. 1C and 4C). This result indicates that the prolonged thermodynamically forced (i.e., SIC-related) BKS warming is sufficient to explain the lack of long-term warming over central Eurasia and help clarify the observed warming hole in winter central Eurasia during the period 1980–2022.

## DISCUSSION

On the basis of the observations and the EDAA, we demonstrate that the observed link between BKS warming and Eurasian cooling is influenced by both atmospheric internal variability and SIC-related thermodynamical changes driven by anthropogenic forcings, but these influences operate on different timescale. During the period 1991–2012, marked by strong central Eurasian cooling on interdecadal timescale, approximately two-thirds of CET cooling is governed by the atmospheric internal variability closely tied to the AO, while the residuals can be attributable to SIC-related thermodynamically forced BKS warming. Although the BKS warming has a weak impact in the CET changes at the interannual scale, the rapid BKS warming during 1991–2012 partially offsets this weak influence, resulting in a detectable contribution to CET cooling during that period. Long-term BKS warming associated with anthropogenic forcings—occurring at a rate three to four times the global average—plays a crucial role in suppressing the externally forced warming in central Eurasia from 1980 to 2022.

Previous studies have primarily used bespoke model experiments to explore the possible climate impacts of Arctic warming (*20*–*25*), but it is reliant on models being able to capture the relevant processes. Our results are based entirely on observational data, thus avoiding reliance on the capabilities of model simulations. Additionally, the effectiveness of our proposed EDAA method has been thoroughly validated, making our conclusions more convincing. Our results support the previous findings that internal variability plays a dominant role in CET changes on interannual and interdecadal timescales (*25*). Notably, our estimate of the causal fraction is slightly larger than that of the previous study (*25*), possibly due to methodological differences or variations in the selection of key regions within the BKS area. Nonetheless, our findings provide an alternative plausible estimate of the causal effect and effectively explain the observed warming hole phenomenon in long-term winter CET changes during 1980–2022. Our results suggest a weak but non-negligible Eurasian cooling response to BKS warming on multidecadal timescales. These findings help to resolve the previous contradictions (*6*–*26*) and emphasize the timescale dependence of the impact of internal variability and forced BKS warming on the Eurasian winter climate. This advances our understanding of the causal relationship between Arctic and mid-latitude linkages and provide insights into the long-term changes in Eurasian winter climate.

Given that the observed long-term BKS warming associated with SIC loss is primarily driven by greenhouse gas forcings, it is reasonable to expect that this cooling effect persists over long timescales if greenhouse gas emissions continue to rise in the future. Internal variability—especially related to the AO—plays a dominant role in the CET changes at interannual and interdecadal timescales. Therefore, its influence on Eurasian winter SAT should receive special consideration in near-term climate projections. Last, while anthropogenic forcings can notably influence SIC changes, the preceding SIC variability may also be affected by internal variability at the time (*44*, *45*). Since this study primarily focuses on how thermodynamically forced BKS warming, closely tied to preceding SIC changes, affects the winter mid-latitude climate, the question of how much of the preceding SIC variability is driven by anthropogenic forcings versus internal variability remains an important area for future investigation.

## MATERIALS AND METHODS

### Observational data

The observational proxies of monthly atmospheric circulation, SAT, and surface THF data were provided by the European Centre from Medium-Range Weather Forecast (ECMWF) Reanalysis v5 from 1979 to present [ERA5; https://www.ecmwf.int/en/forecasts/dataset/ecmwf-reanalysis-v5; (*46*)]. Considering that observation stations in the Arctic region were sparse before the satellite observation era and data representativeness was relatively low, this study thus primarily relies on data collected after 1979. The surface THF is the sum of the surface sensible and latent heat fluxes. The SIC was extracted from the Hadley Centre Sea Ice and Sea Surface temperature dataset [HadISST; https://www.metoffice.gov.uk/hadobs/hadisst/; (*47*)]. All the data are interpolated to a 2.5° by 2.5° grid. Seasonal means were considered throughout this paper, and winter means were constructed by averaging monthly means of December, January, and February (DJF). Here, our convention is that the winter of 1980 refers to the 1979/80 winter. The late autumn/early winter were constructed by averaging monthly means of October, November, and December (OND). To eliminate the noise, all the data are smoothed using 3-year running averages before computing the trend. The statistical significance for the regression and correlation coefficients was evaluated with a two-sided Student’s *t* test.

### Extended dynamical adjustment approach

Dynamical adjustment approach (DAA) has a long history and is originally applied to weather prediction, based on the idea that similar atmospheric circulation patterns exert the similar influences on SAT changes (*48*). Recently, the DAA is widely used to isolate the circulation-induced variability in land SAT that can obscure the thermodynamically induced changes in response to radiative forcing from increasing greenhouse gases (*49*–*53*). The specific steps for DAA based on constructed circulation analogs using sea level pressure (SLP) fields are summarized below: First, we reconstruct the SLP pattern for a given winter using a linear combination of SLP patterns in the same winter from 30 randomly selected years in the dataset (excluding the year in question) based on multiple linear regression. For example, to reconstruct the winter SLP pattern in 1980, we randomly sample 30 selected winters during 1980–2022 and then compute their linear combination through multiple linear regressions to derive a constructed SLP analog that matches the observed winter SLP field of 1980. Using the same weighting coefficients from this reconstruction, we reconstruct the full spatial SAT fields to represent the component associated with atmospheric circulation–induced SAT changes during the observed winter of 1980. This process is repeated 200 times to prevent overfitting. The thermodynamically forced change in SAT for the winter of 1980 is determined by linearly removing the atmospheric circulation–related component, averaged from the 100 reconstructions, from the original SAT variability.

However, the use of DAA to isolate the atmospheric circulation–induced variability has certain limitations in areas with strong air-sea or air–sea ice interactions because the local atmospheric circulation itself may also respond to surface thermodynamic forcing in these regions. Therefore, the isolated circulation-related SAT variability based on DAA using local SLP field actually includes both the SAT change caused by atmospheric circulation and the SAT part that changes the local atmospheric circulation. Figure S5 shows the decomposed BKS SAT variability based on DAA using local SLP field (75°N to 85°N, 15°E to 80°E) in BKS during 1980–2022. We find that the isolated atmospheric circulation–related BKS SAT variability (BKST_Dyn_) is almost equal to the original BKS SAT variability, with the residual port (BKST_Res_) being negligible. However, we know that the thermodynamic processes associated with the anthropogenic forcing have an important impact on the SAT changes in the BKS region (*2*, *3*). Therefore, this result indicates that the thermodynamically forced BKS SAT variability has a definite influence on the local atmospheric circulation changes there because the circulation-related BKS SAT variability isolated by DAA includes the SAT component that alters the local atmospheric circulation, which results in the remaining BKS SAT component being nearly zero. Therefore, in the BKS region, using local SLP for DAA cannot effectively isolate the part where atmospheric circulation affects BKS SAT variability.

To better isolate the atmospheric circulation–induced BKS SAT variability, we propose an extended DAA (EDAA) to explicitly separate BKS SAT changes into components driven by the atmospheric circulation and thermodynamic processes associated with anthropogenic forcing. Considering the potential minor impact of SAT changes with a local region on broader hemispheric atmospheric circulation changes, EDAA involves dynamically adjusting the circulation field over a broader area. Here, we use the hemispherical-scale SLP field in the Northern Hemisphere (50°N to 90°N) as a dynamic factor to separate the atmospheric circulation–induced and thermodynamically forced components of BKS SAT variability. EDAA can reconstruct the original SLP field in BKS region very well—the correlation coefficient between the original and reconstructed area-averaged SLP over BKS reaches 0.99 (fig. S6). To verify the effectiveness of EDAA, the surface THF and preceding SIC changes associated with the decomposed BKS SAT variability are further examined.

Previous studies have reported that the direction of THF anomalies can provide insights into the predominant interactions between SAT and atmospheric circulation (*5*, *25*, *54*–*57*). In Arctic, reductions in sea ice can induce an increase in upward THF at the surface, which warms SAT and can further alter the atmospheric circulation through several mechanisms (*7*, *17*). Conversely, the southerly intrusions associated with large-scale atmospheric circulation are also associated with anomalous downward surface THF and warm the SAT in the Arctic (*54*–*57*). We argue, therefore, that the upward THF anomalies associated with the BKS warming indicate BKS SAT changes driving the atmospheric circulation, while downward THF anomalies suggest the reverse. Here, the negative THF values indicate that the surface gains heat transferred downward from the atmosphere, while positive THF values indicate that the atmosphere gains heat released upward from the surface. Additionally, numerous studies have highlighted that, in addition to winter atmospheric internal variability, BKS SAT changes are largely influenced by surface thermodynamic processes, particularly the strong sea ice–SAT feedbacks associated with anthropogenic forcings (*2*, *3*). Therefore, variations in preceding SIC from late autumn and early winter (OND) can further help elucidate the causality. Therefore, if the circulation-induced BKS warming isolated by EDAA is accompanied by downward THF and no significant connection to the earlier SIC, it can be reasonably concluded that this portion of BKS SAT variability is primarily driven by winter atmospheric circulation changes. On the other hand, if the thermodynamically forced portion of BKS warming is accompanied by upward THF and a significant reduction in preceding SIC, it suggests that this part of BKS SAT variability is primarily driven by thermodynamic forcing, particularly the changes in preceding SIC.

Figure 2 show the original and decomposed BKS SAT variability using EDAA. Here, the atmospheric internally driven BKS SAT variability isolated by EDAA is termed as BKST_Internal_ and the thermodynamically forced part of BKS SAT variability is termed as BKST_Forced_. It is clear that an anomalous anticyclone over the BKS and Ural regions, accompanied by widespread downward THF anomalies in the BKS regions, is associated with the increase in BKST_Internal_ (Fig. 2B). No significant preceding SIC anomalies are observed associated with the increase in BKST_Internal_ (Fig. 2E). This confirms that EDAA can effectively isolate the circulation-induced variability in BKS SAT by extracting it without contamination from the SIC-related thermodynamically forced variability, thereby focusing on the portion driven by atmospheric circulation. This result supports previous findings that winter atmospheric internal variability plays an important role in the Arctic–mid-latitude climate connection during boreal winter (*20*–*26*).

Additionally, we observed that with the increase in BKST_Forced_, there is a significant upward THF anomaly in the core region of BKS, accompanied by significant reduction of preceding SIC (Fig. 2, C and F). Corresponding to this upward THF over BKS, an anomalous anticyclone is also observed over the BKS and Ural regions, but with a relatively weaker magnitude than that generated by atmospheric internal variability (Fig. 2B). This confirms that the thermodynamically forced BKS SAT variability isolated by EDAA is mainly associated with the variation of preceding SIC. This result also supports the previous inference that the thermodynamically forced BKS warming associated with preceding SIC changes can drive an atmospheric circulation response, but with weak magnitudes (*6*–*12*). This can also partly explain the weak atmospheric circulation responses to the SIC loss in the numerical models (*17*, *33*). Note that there are interactions and feedbacks between BKST_Forced_ warming and the atmospheric circulation over various timescales. Therefore, the BKST_Forced_ warming–induced anticyclonic anomalies can further promote BKS warming through the advection of warm air from the south. This feedback process may lead to the formation of downward THF anomalies, countering the upward THF driven by BKS warming. This could explain the presence of downward THF anomalies in the southern part of BKS, where significant southerly anomalies are observed (Fig. 2C).

We have identified that when the SLP field is expanded to the area north of 40°N (from 40°N to 90°N), the effectiveness of EDAA in elucidating the causal relationship between BKS SAT variability and atmospheric circulation changes remains almost unchanged (fig. S7). The correlation coefficients between the BKST_Internal_ indices and using different SLP fields all exceed 0.95 and exceed 0.98 in BKST_Forced_ indices (fig. S7, A and B). To avoid potential interference from low latitude, EDAA is conducted using the SLP field north of 50°N in this study. Additionally, EDAA is also not sensitive to the precise choice of the number of repetitions and the results are less spread when the random samples are more than 25. The correlation coefficients using different random samples all exceed 0.98 in BKST_Internal_ and BKST_Forced_ indices (fig. S8).

### Definitions of BKST and CET indices

The area-averaged SAT in the region with the largest warming over BKS (75°N to 85°N, 15°E to 80°E) is used to represent the BKS SAT variability, denoted as BKST, and the winter atmospheric circulation–induced and thermodynamically forced components (i.e., SIC-related) are denoted BKST_Internal_ and BKST_Forced_, respectively. Note that in this study, BKST_Forced_ variability is not obtained by directly subtracting BKST_Internal_ from BKST, as the BKST_Forced_ derived this way would not be independent of BKST_Internal_ and would exhibit a significant negative correlation. Therefore, we used a linear regression method to remove the BKST_Internal_ variability from original BKST to obtain the BKST_Forced_ component. The CET index is defined as the area-averaged winter SAT over central Eurasia (40°N to 65°N, 65°E to 130°E). The CET index independent of BKS warming is defined by removing the BKST variability from the CET variability through linear regression, denoted as CET_RM_BKST_.
