# Supplementary material for: Teleconnection from Arctic warming suppresses long-term warming in central Eurasia
Source: Sci Adv. 2025 Mar 19;11(12):eadq9461. doi: 10.1126/sciadv.adq9461 (PMC11922007; doi:10.1126/sciadv.adq9461)
Supplement: Supplementary file 1 — Figs. S1 to S8 [file sciadv.adq9461_sm.pdf]

Science Advances
AAAS

Supplementary Materials for

Teleconnection from Arctic warming suppresses long-term warming in central Eurasia

Hainan Gong *et al.*

Corresponding author: Lin Wang, wanglin@mail.iap.ac.cn; James A. Screen, j.screen@exeter.ac.uk; Wen Chen, chenwen-dq@ynu.edu.cn

Sci. Adv. **11**, eadq9461 (2025)
DOI: 10.1126/sciadv.adq9461

This PDF file includes:

Figs. S1 to S8

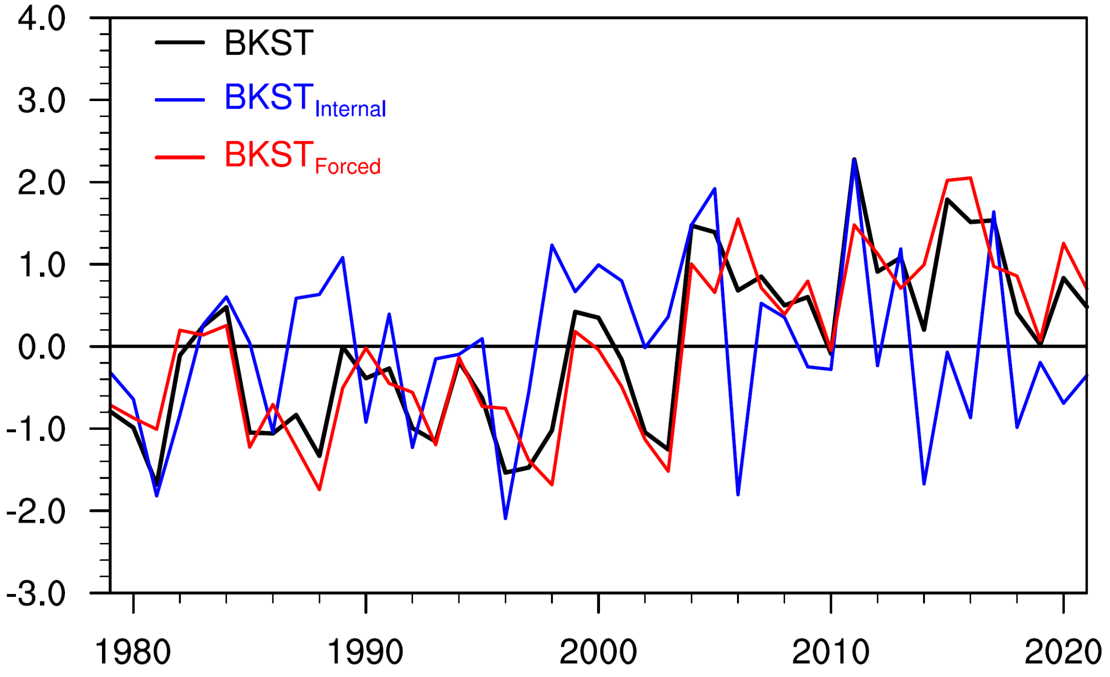

fig. S1. Temporal evolutions of the original and decomposed SAT indices in the BKS. Normalized time series of original and decomposed $BKST_{Internal}$ and $BKST_{Forced}$ indices during 1980-2022.

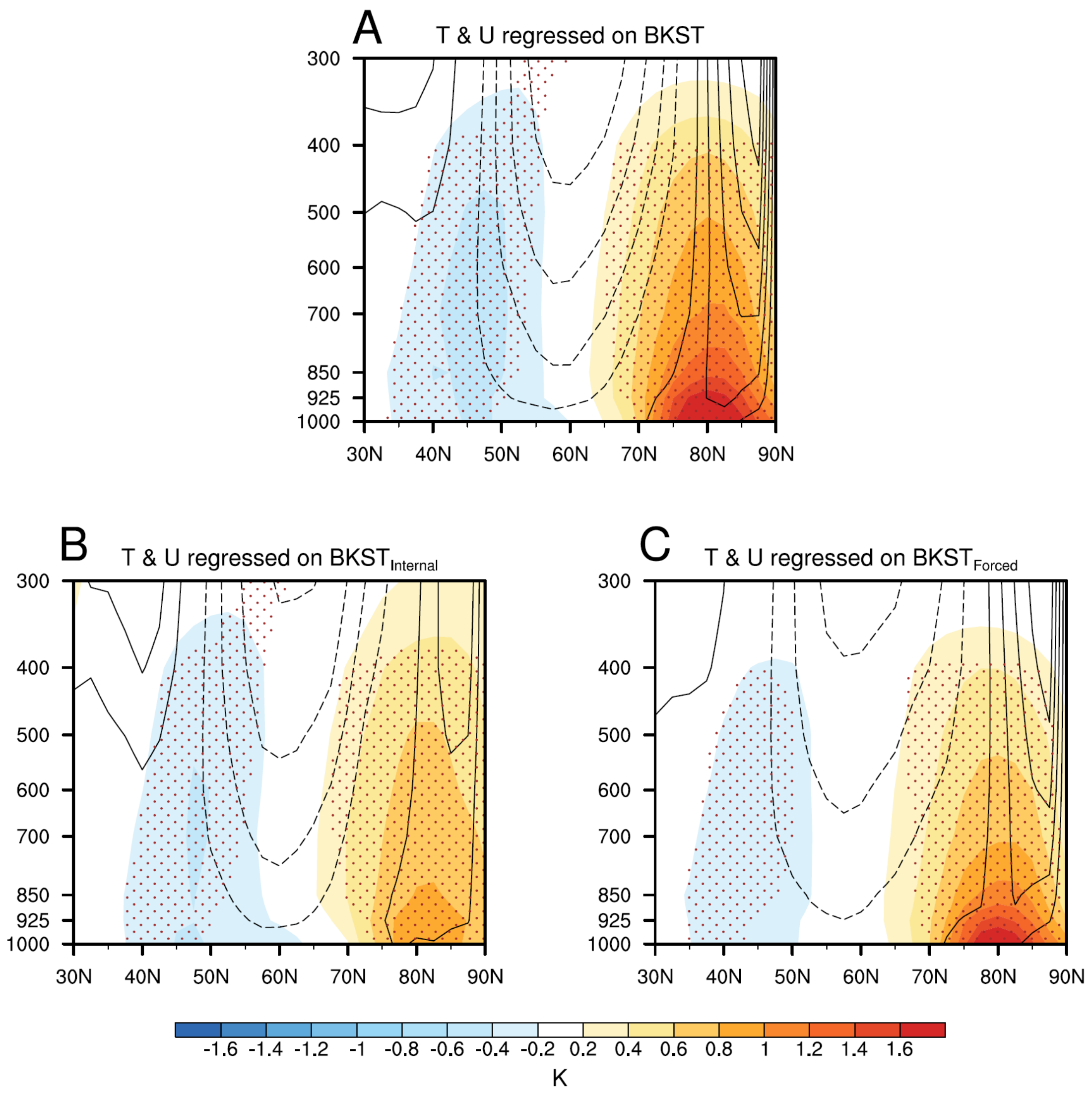

fig. S2. Changes in winter zonal-mean atmospheric circulation and temperature associated with the original and decomposed SAT variability in the BKS. A, Winter zonal-mean temperature (shading) and zonal wind anomalies (contours, 0.3m s -1 interval) along 0°-150°E regressed on the BKST index. **B**, **C**, As in a, but for $BKST_{Internal}$ and $BKST_{Forced}$ indices, respectively during 1980-2022. Dots indicate regions of temperature changes exceeding the 95% confidence level.

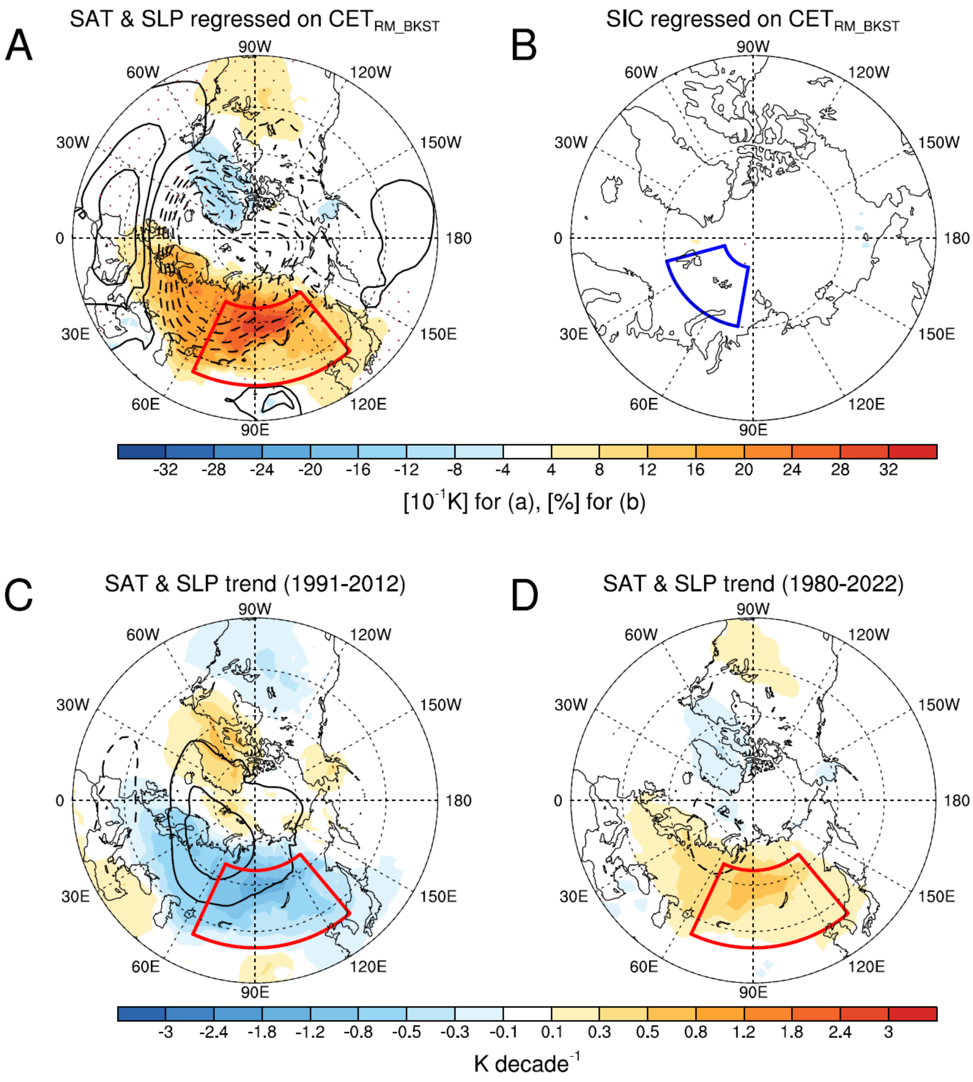

fig. S3. Changes in SAT, atmospheric circulation, and preceding SIC associated with the BKS warming-independent CET variability. A, Winter SAT (shading) and SLP (contours, 0.6hPa interval) anomalies regressed on the normalized CET$_{RM_BKST}$ index during 1980-2022. **B**, As in a, but for the preceding late-autumn/early winter (October to December) SIC anomalies over Arctic. **C**, CET$_{RM_BKST}$ -related winter SAT (shading) and SLP (contours, 0.8hPa per decade interval) trends over the Northern Hemisphere in the period 1991-2012. **D**, As in **C**, but for the period 1980-2022. Dots in (**A**) and (**B**) indicate regions of SAT and SIC anomalies exceeding the 95% confidence level.

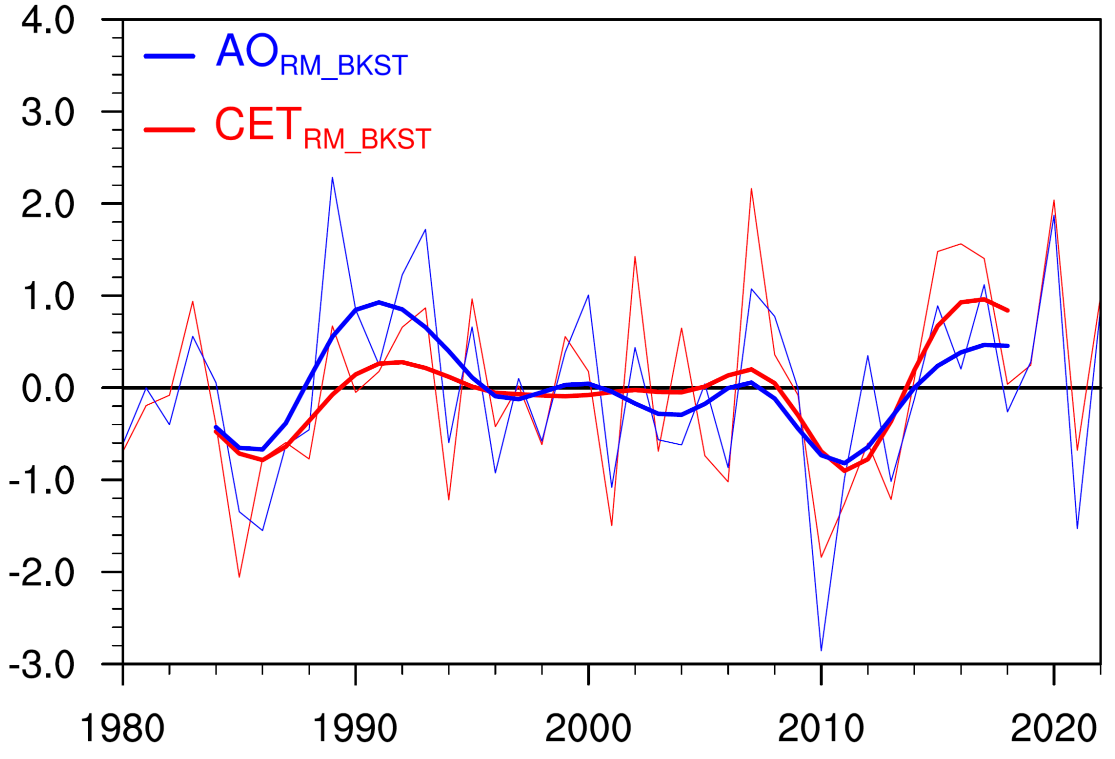

fig. S4. Temporal evolutions of the BKS warming-independent AO and CET indices. Time series of AO_{RM_BKST} and CET_{RM_BKST} indices in the period 1980-2022. The 9-year low-pass components are shown with the corresponding solid lines.

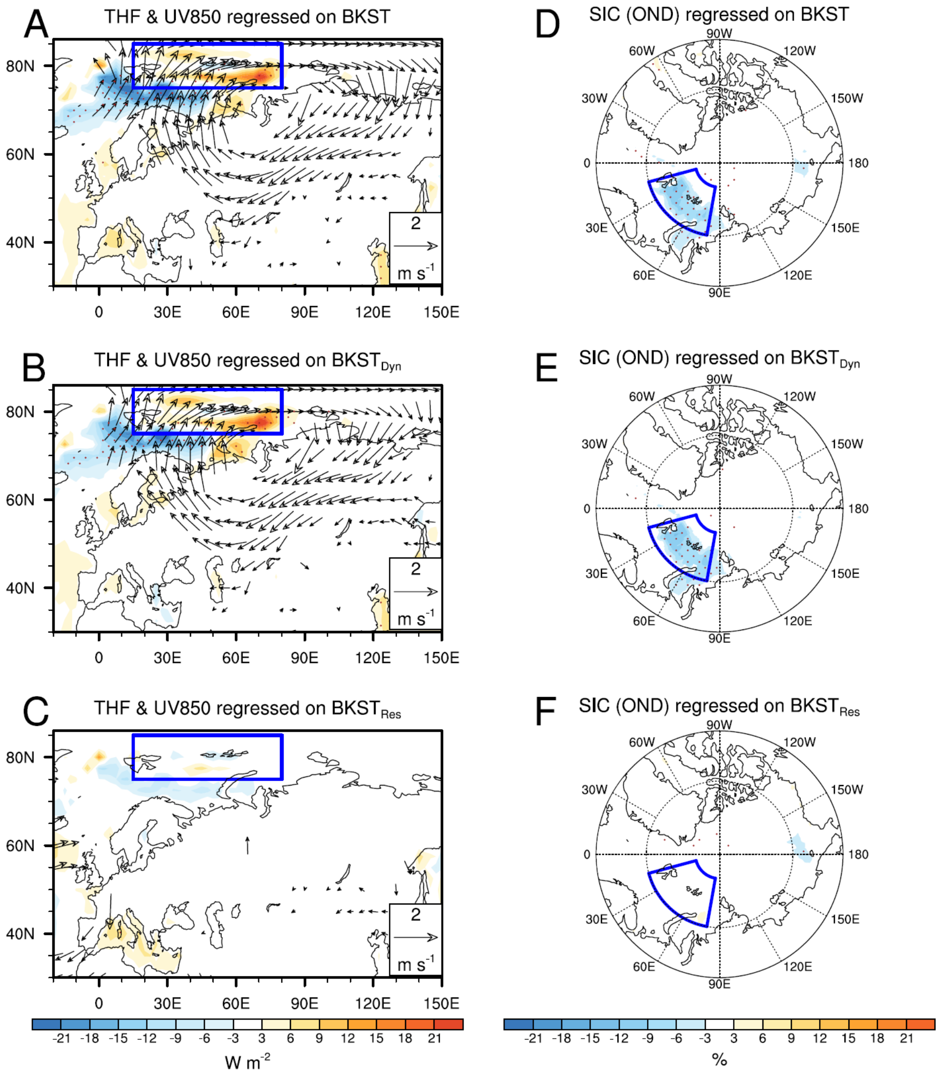

fig. S5. Observed changes in 850hPa wind, turbulent heat flux (THF), and sea ice concentration (SIC) associated with the original and decomposed SAT variability using DAA. A, Winter 850hPa wind (vectors) and THF (shading) anomalies regressed on the BKST index. **B**, **C**, As in **A**, but for circulation-related BKST ($BKST_{Dyn}$) and residual BKST ($BKST_{Res}$) indices, respectively during 1980-2022. **D** to **F** As in **A** to **C**, but for the preceding late-autumn/early winter (October to December) SIC anomalies over Arctic. Dots indicate the regions of THF and SIC changes exceeding the 95% confidence level. The wind vectors are shown only when they exceed the 95% confidence level. The BKS region is indicated by blue box.

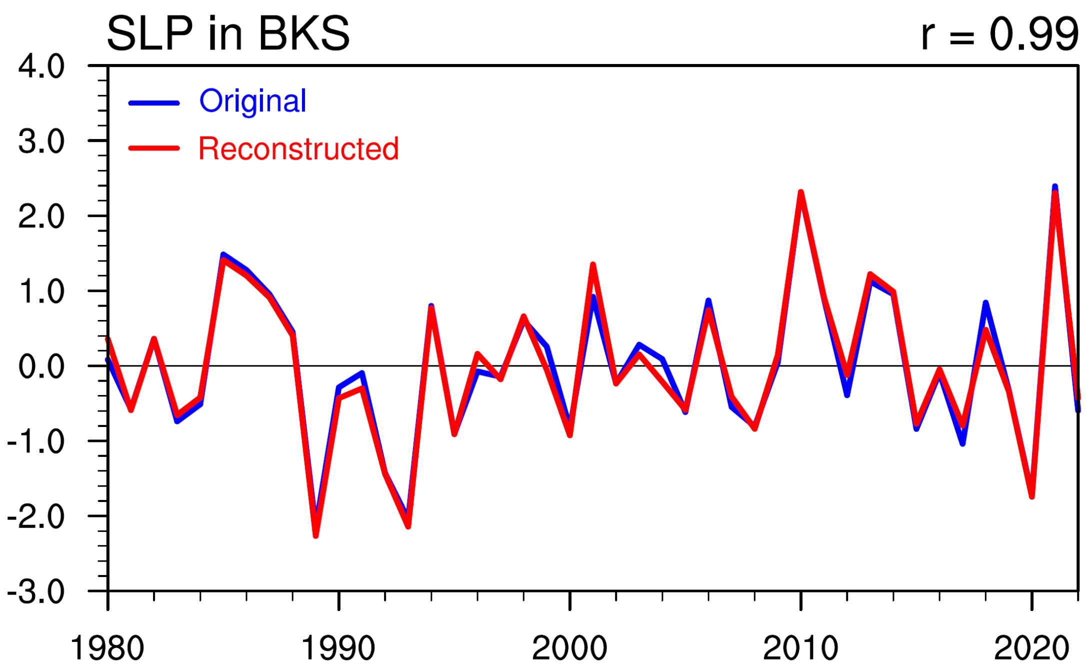

fig. S6. Original and reconstructed SLP in BKS. Temporal evolution of normalized area-averaged SLP over BKS (75°-85°N, 15°-80°E) region in ERA5 (blue line) and reconstructed SLP data obtained using EDAA (red line) during 1980-2022.

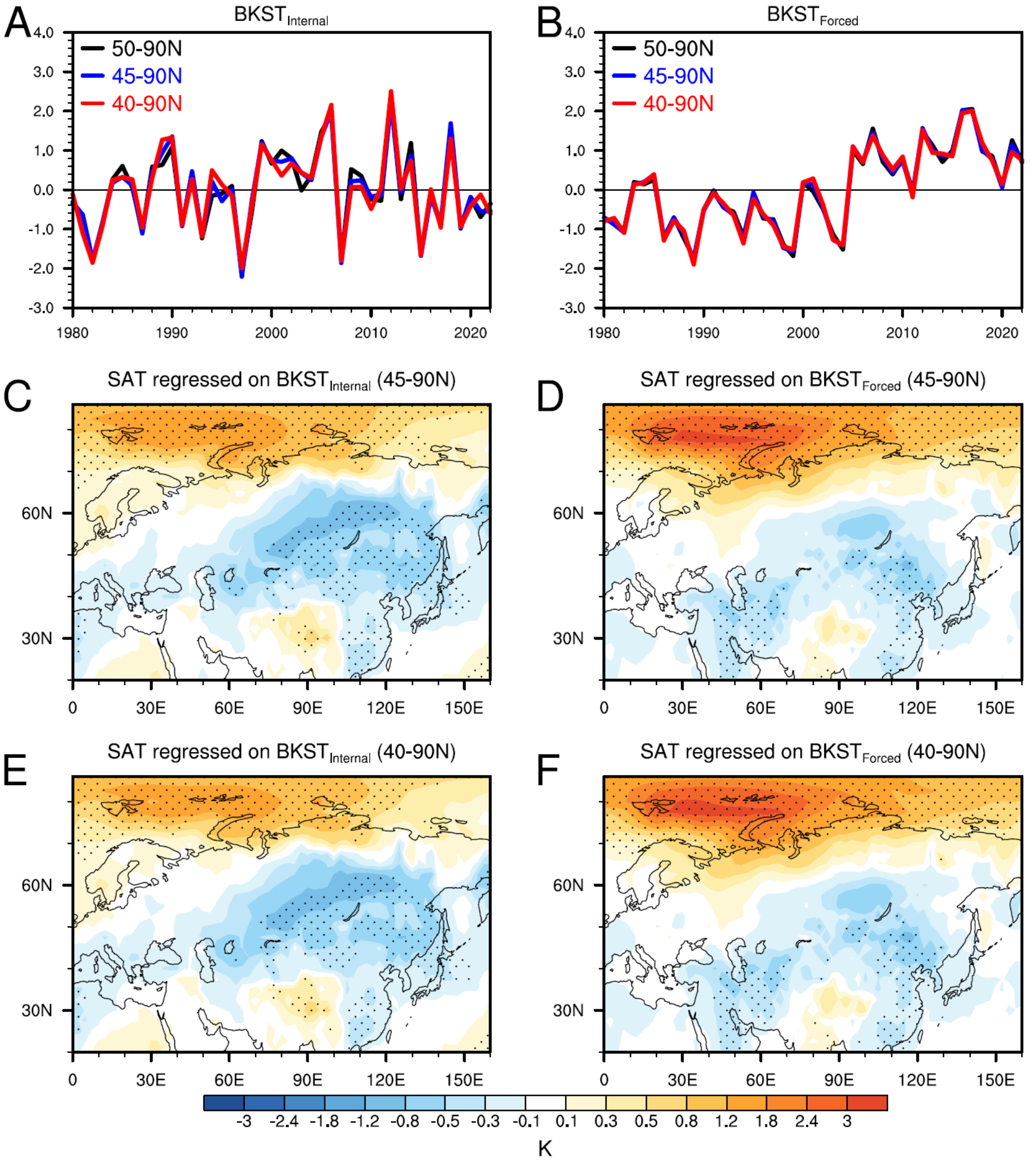

fig. S7. SAT changes associated with the atmospheric internally-induced and thermodynamically-forced BKST variability obtained using different range of SLP analogs in EDAA. Time series of normalized $BKST_{Internal}$ (**A**) and $BKST_{Forced}$ (**B**) indices during 1980-2022 obtained from different range of SLP analogs in EDAA. The SAT anomalies regressed on the $BKST_{Internal}$ index obtained using 45°-90°N SLP analogs (**C**) and 40°-90°N SLP analogs (**E**) analogs. **D**, **F** As in **C**, **E**, respectively, but for the $BKST_{Forced}$ index. Dots indicate SAT anomalies exceeding the 95% confidence level.

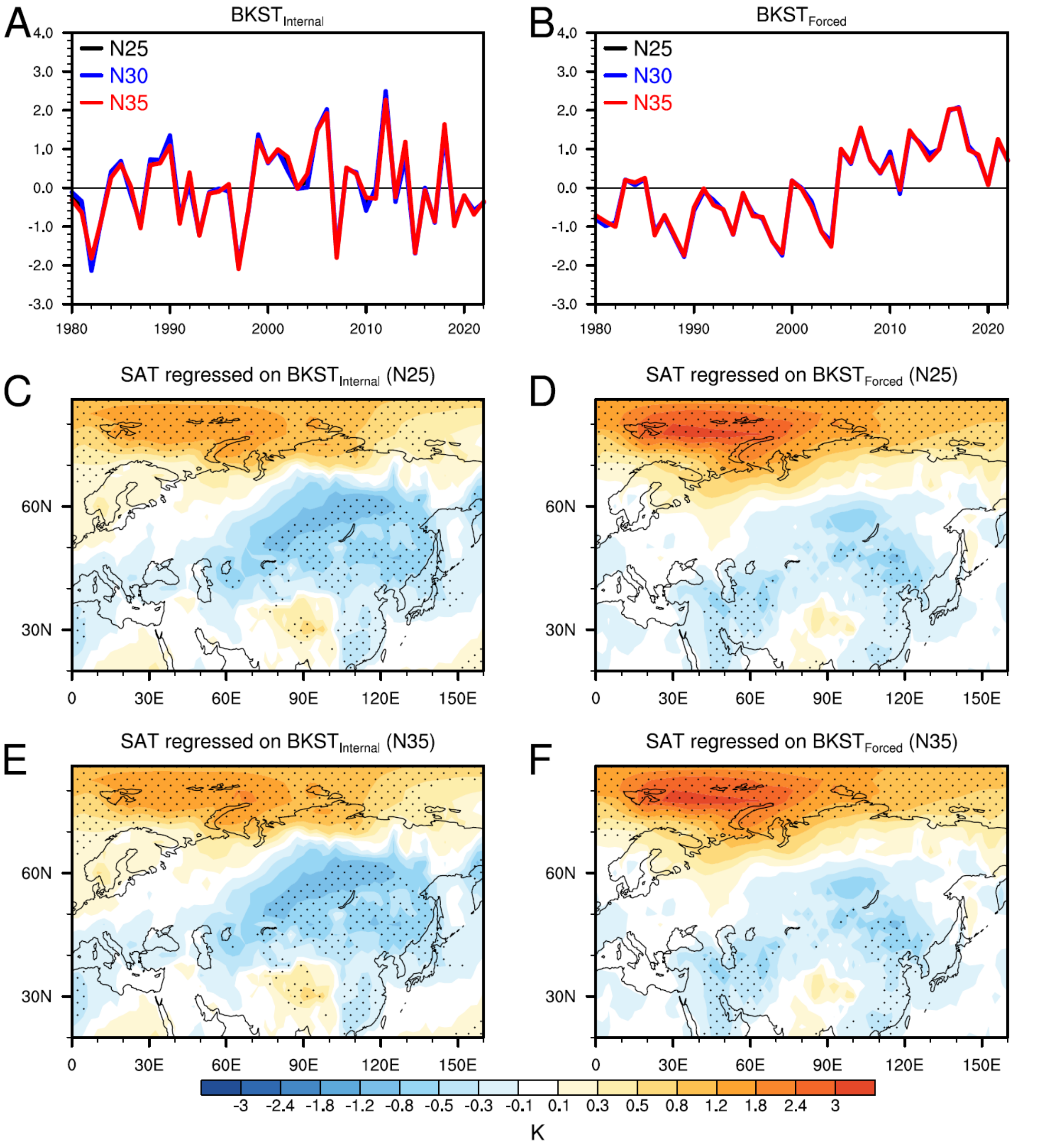

fig. S8. SAT changes associated with the atmospheric internally-induced and thermodynamically-forced BKST variability obtained using different numbers of SLP analogs in EDAA. Time series of normalized BKST$_{\text{Internal}}$ (**A**) and BKST$_{\text{Forced}}$ (**B**) indices during 1980-2022 obtained using different numbers of SLP analogs in EDAA. The SAT anomalies regressed on the BKST$_{\text{Forced}}$ index obtained based on 25 (**C**) and 35 (**E**) 50°-90°N SLP analogs. **D**, **F** As in **C**, **E**, respectively, but for the BKST$_{\text{Forced}}$ index. Dots indicate SAT anomalies exceeding the 95% confidence level.
